# Late Hybrid Retrieval of an Embolized Left Atrial Appendage Occlusion Device: A Case and Literature Review

**DOI:** 10.1016/j.jaccas.2026.106937

**Published:** 2026-02-11

**Authors:** John P. Birrane, Michael Killian, Gregory Offiah, Ross T. Murphy, Sandra Quinn, Prakash Madhavan, Ignacio Cruz-Gonzalez, Kevin P. Walsh, Andrew O. Maree

**Affiliations:** a Department of Cardiology, St James's Hospital, Dublin, Ireland; b Naas General Hospital, Naas, Ireland; c The Institute of Cardiovascular Science, Trinity College Dublin, Dublin, Ireland; d St James's Vascular Institute, St James's Hospital, Dublin, Ireland; e University Hospital of Salamanca, Salamanca, Spain; f Children's Health Ireland at Crumlin, Dublin, Ireland

**Keywords:** atrial fibrillation, imaging, occluder, stroke, x-ray fluoroscopy

## Abstract

**Background:**

Device embolization after left atrial appendage occlusion (LAAO) is rare (∼0.1%) but may be life-threatening.

**Case Summary:**

A 72-year-old man presented with a left frontal stroke 2 years after Amplatzer Amulet LAAO device implantation. Computed tomography angiography showed the occluder lodged across the left subclavian artery ostium in the transverse aortic arch. After heart team review and Health Products Regulatory Authority compassionate-use approval, an ONO basket retrieval system (Onocor) was used to capture the device under fluoroscopic and transesophageal echocardiography guidance. Repeated attempts to resheath it into a 26-F DrySeal sheath failed because the device could not be adequately compressed. The device was withdrawn to the right common iliac artery, where evolving limb ischemia prompted surgical device extraction via an infrarenal aortotomy. Extensive tissue ingrowth was evident on device retrieval.

**Discussion:**

Review of 18 published reports describing 20 cases of late LAAO device embolization showed retrieval intervals up to 18 months, almost all successfully managed percutaneously. This case extends that experience to 2 years and highlights how chronic tissue incorporation can hinder resheathing, emphasizing the role of hybrid retrieval strategies when percutaneous extraction fails.

**Take-Home Messages:**

Embolized LAAO devices that remain in situ for prolonged periods may develop tissue ingrowth, making percutaneous removal technically challenging. When full percutaneous extraction is not achievable, hybrid percutaneous-surgical retrieval via an infrarenal aortotomy offers a safe bailout that avoids sternotomy.

## Visual Summary

**Figure:**
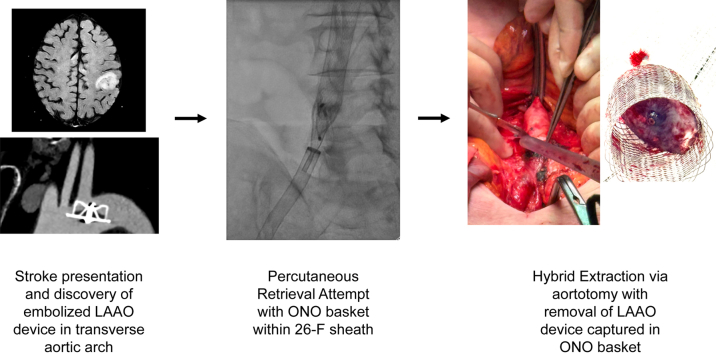

Embolization of left atrial appendage occlusion (LAAO) devices is a rare complication, occurring in approximately 0.1% of cases.^1^

**Take-Home Messages**

• Embolized LAAO devices that remain in situ for prolonged periods may develop tissue ingrowth, making percutaneous removal technically challenging.
• When complete percutaneous retrieval is not achievable, a planned hybrid percutaneous-surgical approach offers a safer alternative to sternotomy and cardiopulmonary bypass.

## History of Presentation

A 72-year-old man was admitted with an episode of transient dysarthria and right-hand weakness. Brain magnetic resonance imaging confirmed an acute posterior left frontal lobe stroke. His past medical history included atrial fibrillation treated with pulmonary vein isolation ablation and implantation of a 25-mm Amulet LAAO device (Abbott) in 2023 in another jurisdiction. The patient received direct current cardioversion for atrial fibrillation 3 weeks after LAAO device implantation.

## Investigations

**Figure 1:**
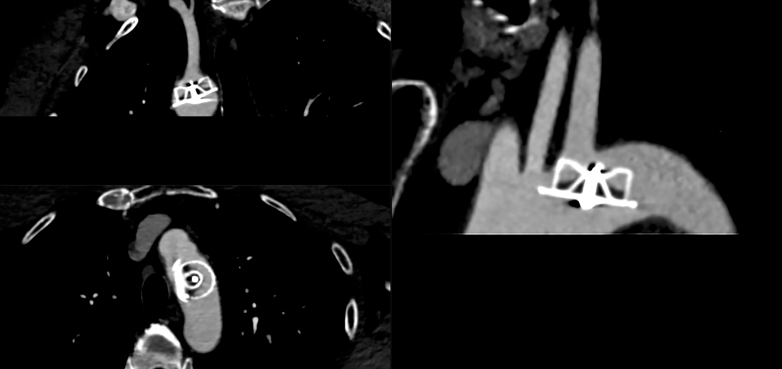
Computed Tomography Angiogram of Aortic Arch Computed tomography angiogram demonstrated the presence of the embolized Amulet left atrial appendage occlusion device in the transverse aorta at the left subclavian artery ostium.

An LAAO device was not visualized on echocardiography, which otherwise showed a structurally normal heart. Computed tomography angiography of the aortic arch revealed the LAAO device in the transverse aortic arch, across the origin of the left subclavian artery (Figure 1).

The case was discussed and imaging reviewed at our institutional heart team meeting. Given the patient's recent stroke and the obstructive location of the LAAO device, we decided that device retrieval was required. Owing to the device's location in the transverse aorta, we decided to attempt percutaneous retrieval in the first instance with open surgical retrieval as a bailout option, for which the patient was specifically consented. The heart team concluded that the device had most likely embolized shortly after implantation and had remained in situ for approximately 2 years. To prevent secondary embolization of the device during withdrawal, retrieval using the ONO basket retrieval device (Onocor) was planned. The device was approved on compassionate-use grounds by the Health Products Regulatory Authority given the absence of a CE mark for use in Europe.

## Management

The procedure was performed under general anesthesia in a hybrid cardiac catheterization laboratory with operating room capability. A cardiothoracic surgeon was present and a perfusionist was on standby in case of thoracic aortic injury requiring surgical conversion. Bilateral common femoral arterial access was obtained with ultrasound guidance. The right common femoral artery access was preclosed with 2 Perclose ProStyle suture systems (Abbott) and then serially dilated with 12-F and 18-F dilators. A 26-F DrySeal Flex introducer sheath (Gore Medical) was inserted over an Amplatz Super Stiff guidewire (Boston Scientific). The patient received intravenous heparin to achieve an activated clotting time of >200 seconds.

**Figure 2:**
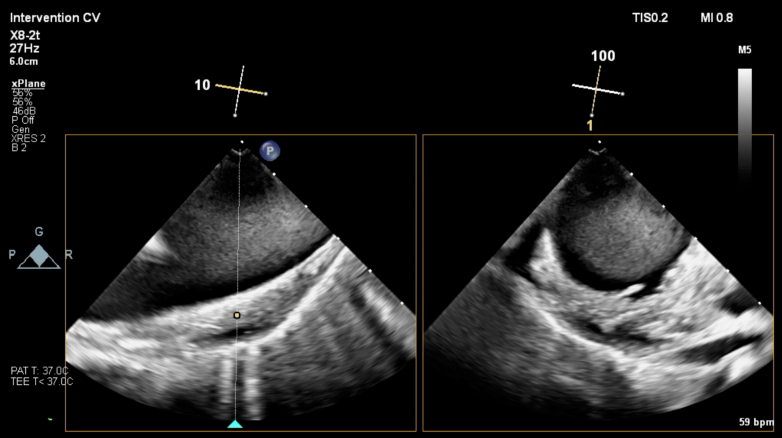
Intraprocedural Transesophageal Echocardiography Image Transesophageal echocardiography showing intramural hematoma after device dislodgement from the transverse aorta.

**Figure 3:**
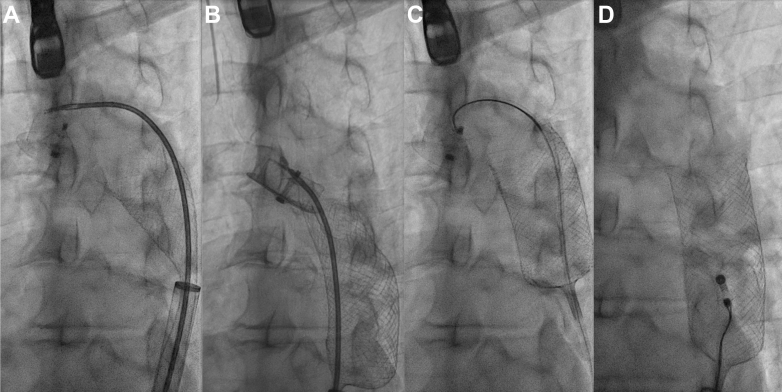
Fluoroscopic Images Demonstrating Amulet Device Within the ONO Basket in the Aortic Arch (A and B) An initial attempt was made to secure the embolized Amulet with a Raptor grasping device, but there was difficulty in getting the device coaxially aligned with the device. (C and D) A 15-mm gooseneck snare was used to grasp the button on the Amulet, allowing it to be withdrawn into the ONO basket. The encaged Amulet was withdrawn to the right common femoral artery, where further attempts to compress and reorient it for withdrawal into the 24-F DrySeal sheath were unsuccessful.

The sheath was advanced to the transverse aorta, and the ONO basket retrieval system was positioned underneath the LAAO device. Under direct fluoroscopic and transesophageal echocardiographic guidance, a Raptor rat-toothed forceps (Steris Healthcare) was advanced through the ONO basket and grasped tissue within the LAAO but could not be aligned coaxially with the device and was unable to disengage it from the wall of the transverse aorta (Figure 2). A 15-mm gooseneck snare was advanced through a 6-F JR4 guide catheter and was used to snare the distal end screw on the lobe of the LAAO and successfully retrieve it into the ONO basket (Video 1). A small intimal tear and localized intramural hematoma in the transverse aorta was visualized on transesophageal echocardiography and was monitored continuously for stability throughout the remainder of the procedure (Figure 3).

**Figure 4:**
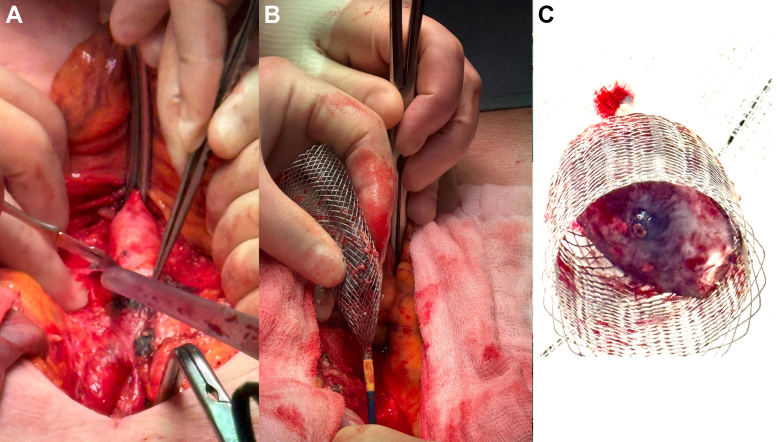
Open Retrieval of Embolized Amulet Device Within ONO Basket After withdrawal of the embolized Amulet within the ONO basket to the right common femoral artery, evolving limb ischemia prompted urgent vascular surgery consultation. (A and B) Vascular surgery proceeded to a mini midline laparotomy, where the device was extracted through the infrarenal abdominal aorta. (C) The device was heavily infiltrated with tissue and adherent thrombus, which likely accounted for the difficulty in compressing and removing the device through an entirely percutaneous route.

Repeated attempts were made with a Raptor rat-toothed forceps, gooseneck snares, and a Bioptome biopsy forceps to collapse the LAAO device into the DrySeal sheath but were unsuccessful. The LAAO device within the ONO basket was withdrawn to the right common iliac artery ostium, where further attempts were made to maneuver the recaptured device into the sheath (Figure 2, Video 2). We concluded that given the device size, time since implantation, and likely incorporation with tissue, further constraint of the device into the sheath was unlikely to be successful. In the interim, the right lower limb had become cool, without palpable pulses. After conferring with vascular surgery, the DrySeal sheath was withdrawn from the common femoral artery and the ProStyle sutures synched down on the ONO delivery system. We proceeded to perform open vascular retrieval of the LAAO device and ONO system via laparotomy. The infrarenal abdominal aorta was cross-clamped, and the LAAO device and ONO system were extracted via abdominal aortotomy (Figure 4). Inspection of the LAAO device revealed extensive infiltration of soft tissue.

The patient was transferred to the intensive care unit, where he made an uneventful recovery. A computed tomography aortogram was carried out to assess the intramural hematoma caused by device dislodgement from his transverse aorta and was found to be stable with no significant dissection.

## Discussion

Large case series and multicenter registries report that the majority of LAAO device embolization (60%-70% of cases) occurs within 24 hours of implantation.^2^^,^^3^ Late embolization was defined in the only large registry of embolized LAAO devices as that occurring >45 days after implant.^2^ The aorta is the most common site of device embolization and presents the lowest risk for transcatheter retrieval.^1^ Risk factors for device embolization include device missizing, shallow left atrial appendage anatomy, and changes in rhythm between atrial fibrillation and sinus rhythm. The latter is notable given that our patient received direct-current cardioversion for atrial fibrillation 3 weeks after device implantation. The restoration of left atrial contractility prior to complete device endothelialization is a plausible trigger for embolization.

To our knowledge, this case details the longest interval (2 years) between implantation and successful retrieval published in the literature. We conducted a focused search of PubMed and Google Scholar for published case reports and series detailing percutaneous retrieval of late embolization of LAAO devices to try and identify techniques and challenges related to late retrieval. We chose the cutoff of >45 days proposed by Eppinger et al^2^ in their large, dedicated registry for embolized LAAO devices because we felt that this interval was most likely to reflect the challenges faced in late retrieval cases such as ours. A total of 18 published reports describing 20 cases of late LAAO device embolization were identified (Supplemental Table 1). Retrieval intervals ranged from 6 weeks to 18 months after implantation. The aorta and its branches represented the most frequent sites of device lodgment, most commonly the abdominal aorta, while several reports described left atrial or left ventricular outflow tract migration. Most patients were asymptomatic at the time of detection, though stroke, dyspnea, or distal ischemia occasionally prompted investigation. Patients with devices embolized to the left ventricular outflow tract were more likely to have symptoms, whereas only 1 case of embolization to the aorta was associated with clinical signs or symptoms.^4^

Percutaneous retrieval was successful in most cases, usually via femoral arterial access, and employed large-bore sheaths (16-F to 26-F) and a combination of snares, forceps, and steerable guide catheters. Building on recent first-in-human reports of LAAO device removal using the ONO basket retrieval system, the present case demonstrates successful arch containment of an embolized LAAO device with that system.^5^ Retrieval within a basket device has the advantage of containing the device and preventing further thrombus embolization after dislodgement.^6^, ^7^, ^8^

Several factors complicated our percutaneous retrieval efforts. Prolonged apposition to the transverse aorta after device embolization likely led to extensive endothelialization and incorporation into the intima of the transverse aorta, such that traction on the device during retrieval caused intimal injury and development of an intramural hematoma. In cases of very late embolization, this process should be anticipated preprocedurally, with adequate preparation made for possible conversion to an open surgical or hybrid retrieval. A proposed relationship between prolonged time in situ and more extensive tissue infiltration was supported by soft tissue observed within the device (Figure 4), rendering it relatively incompressible within the ONO basket. A case series by Turagam et al.^9^ noted organized soft tissue within an Amulet in 1 patient at 3 months. They were unable to retract the device into a 12-F deflectable catheter and were required to upsize to a 23-F Micra sheath.

Had we been successful in compressing the device sufficiently to maneuver it into the DrySeal sheath, it is possible the system could have been removed entirely percutaneously. While we felt the larger internal lumen of the DrySeal sheath gave us the best chance of recovering the device, the lack of directionality that is possible with a steerable guide sheath meant that our grasping device and the ONO sheath were not coaxial, which may have been responsible for the failure of our initial engagement. Our eventual technique for capturing the Amulet by using a loop snare to capture the distal end screw is a technique that was successfully used in previous cases.^1^^,^^6^^,^^10^

Although complete percutaneous extraction was not achievable, the hybrid approach, combining endovascular capture with surgical removal via aortotomy, avoided the need for sternotomy and cardiopulmonary bypass. This strategy substantially reduced operative risk, procedural complexity, and recovery time. With appropriate planning, a combined percutaneous and vascular surgical strategy may expand treatment options for late device embolization and avoid the morbidity of open cardiac surgery.

**Visual Summary:**
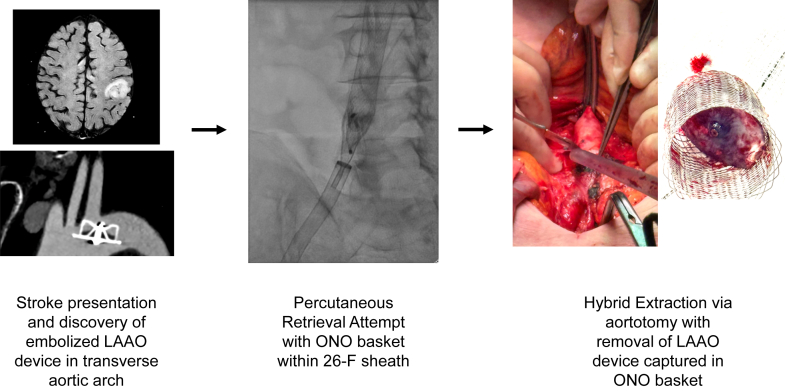
Late Embolization of an LAAO Device With Hybrid Retrieval A 72-year-old man presented with an ischemic stroke 2 years after LAAO implantation. CT angiography identified the device lodged across the left subclavian artery ostium in the transverse aortic arch. Percutaneous capture was achieved using a basket retrieval system under fluoroscopy and TEE guidance, but chronic tissue ingrowth prevented resheathing into a large-bore sheath. A hybrid bailout with infrarenal aortotomy enabled safe extraction, avoiding sternotomy and cardiopulmonary bypass. CT = computed tomography; LAAO = left atrial appendage occlusion; TEE = transesophageal echocardiography.

## Funding Support and Author Disclosures

The authors have reported that they have no relationships relevant to the contents of this paper to disclose.

## Appendix

Video 1 Retrieving the LAAO Device The end screw of the LAAO device is snared by a gooseneck snare advanced through a JR4 guide and withdrawn into the ONO basket.

Video 2 Attempts to Maneuver the Recaptured LAAO Device Into the DrySeal Sheath Repeated attempts to resheath the LAAO device captured within the ONO basket were unsuccessful.

Supplemental Table 1 Case Reports Describing Percutaneous Management of Late Embolization of LAA Closure Devices.
