# Supplementary material for: Late Hybrid Retrieval of an Embolized Left Atrial Appendage Occlusion Device: A Case and Literature Review
Source: JACC Case Rep. 2026 Feb 11;31(11):106937. doi: 10.1016/j.jaccas.2026.106937 (PMC13008532; doi:10.1016/j.jaccas.2026.106937)
Supplement: Supplemental Table 1 — Case Reports Describing Percutaneous Management of Late Embolization of LAA Closure Devices. [file mmc3.docx]

**Supplemental Table 1. Case reports describing percutaneous management of late embolization of left atrial appendage closure devices**

| **Authors**  **(Year)** | **Clinical presentation** | **Device**  **(Size)** | **Embolization location** | **Retrieval Interval** | **Access** | **Cerebral protection** | **Tools used** | **Technique** | **Complications** |
| --- | --- | --- | --- | --- | --- | --- | --- | --- | --- |
| Siordia et al.^1^  (2025) | 70M, asymptomatic, discovered on routine post-implant TEE | Amulet (25mm) | Transverse aortic arch | 45 days | Right common femoral artery (CFA), 18-F Gore Dryseal | None | 14-F double-curved Amulet sheath (Abbott)  7-F JR4 guide  Ensnare 18/30mm (Merit Medical)  ONO basket retrieval device (ONOCOR) | Device snared and after failed retrieval into 14-F sheath, the ONO basket, together with a snare, was used to capture, compress and retrieve the device | None |
| Guddeti et al. ^2^  (2025) | 68F, asymptomatic, discovered on TTE investigating aortic stenosis | Watchman FLX (31mm) | Left atrium (LA) | 1 year | Bilateral common femoral vein (CFV) access | None | 17-F Bayliss VersaConnect and TruSteer catheter  20-F DrySeal sheath  ONO retrieval system  2.4-mm Raptor forceps (Steris) | After transseptal access to the left atrium, a steerable sheath was used to position the ONO basket over the device, through which it was captured and retrieved | None |
| Green et al.^3^  (2025) | 74M, asymptomatic, discovered incidentally on fluoroscopy during spinal surgery | Amulet (27mm) | Abdominal aorta | 2 months | Right CFA | None | Perclose Prostyle systems  22-F Dryseal Flex sheath (Gore)  4-F USL Shepard’s hook-style catheter (Cordis Medical)  Benson guidewire  7-F Atrieve vascular snare (Argon Medical)  Laparoscopic biopsy grasper | A Benson guidewire was advanced through the device and then snared, forming a closed loop. The device was retracted to the aorta, after which a laparoscopic biopsy grasper was used to grasp the platinum threads on the device and retrieve it into the 22-F sheath. | Subcapsular hematoma involving the superolateral  aspect of the right kidney causing abdominal pain.  Managed conservatively and was discharged postoperative day 5. |
| Goodyear et al.^4^  (2025) | 77F, asymptomatic, discovered on routine post-implant TEE | Watchman FLX (24mm) | Left ventricular outflow tract (LVOT) | 45 days | Right CFV  Right CFA 20-F Cook sheath | Attempted placement of bilateral Sentinel (Boston Scientific) but abandoned due to severe tortuosity in the brachiocephalic artery and left subclavian artery occlusion | Agilis NxT Steerable sheath (Abbott)  Amplatz-Extrastiff wire  40cm 20-F Cook sheath (Cook Medical)  8-F shuttle sheath (Cook Medical)  5F Diagnostic Amplatz Left (AL)-1  Rat tooth and alligator grasping forceps (Boston Scientific) | A shuttle sheath was advanced through the aortic valve into the LV. A rat-toothed/alligator forceps was used to grasp the Watchman and withdraw it into the descending aorta. A second forceps was used to stabilize the device and withdraw it into the sheath. | None |
| Nienabar et al.^5^  (2024) | 83M presented with progressive dyspnea and vertigo | Watchman (24mm) | LVOT | 6 months | Left axillary artery 20-F Gore DrySeal | Sentinel | 7-F Raptor grasping device (Steris Healthcare)  ONO basket retrieval device  Destino twist deflectable steerable 13.8-F guiding sheath (Oscor Medical devices) | Device grasped with Raptor forceps and retrieved into ONO, which successfully compressed and captured device into sheath. | None |
| Wong et al.^6^  (2024) | 69M, asymptomatic, discovered on routine post-implant TEE | Watchman FLX (31mm) | LA | 10 weeks | CFV | Sentinel | 24-F MitraClip steerable guide catheter (SGC)  14-F sheath  STERIS raptor forceps  35mm Gooseneck snare | The SGC was advanced transeptally. A Raptor grasping forceps was used to capture the device and withdraw it into the SGC for removal. | On 30-day clinical follow-up, the patient had  returned to work but under permanent modified desk  duty |
| Ferraris et al ^7^  (2022) | 75M with symptoms of dyspnoea and fatigue. Discovered on routine post-implant TEE | Watchman FLX (27mm) | LA | 3 months | Right CFV | None | 2 steerable introducers 8-F, 12-F  Bioptome  Osypka Lasso snare  2nd Snare catheter | Following transseptal access, two snare catheters were used to encircle the device and withdraw it into the 12-F introducer | No complications at 3-month follow-up |
| Ćwiek-Rębowska et al ^8^  (2021) | 67M, incidentally discovered on routine echocardiography | Amulet (22mm) | Abdominal aorta | 8 weeks | CFA | None | Maslanka grasping forceps  MultiSnare | The dislocated device was removed using Maslanka grasping forceps and the MultiSnare via the femoral artery. | None |
| Agarwal et al.^9^  (2021) | 88M, symptoms NR, discovered on 6-week TEE | Watchman (30mm) | LVOT | 6 weeks | 7-F Left CFA  24-F femoral venous | None | 24-F Mitraclip sheath  Raptor grasping device  7-F destination sheath | The destination sheath was advanced from left CFA access through the aortic valve to push the Watchman into the LV. A Mitraclip sheath was advanced to the left atrium through transseptal access. A Raptor grasping device was advanced through the Mitraclip sheath to retrieve the Watchman from the LV. | None |
| Maan et al.^10^  (2021) | 84M, symptoms NR, discovered on echocardiography | Watchman (24mm) | Decompression and protrusion outside left atrial appendage | 7 months | 23-F CFV | 27mm Watchman in Ascending Aorta for Stroke Prevention (WAASP) technique | 23-F Mitraclip sheath  Raptor grasping device  12-F FlexCath Advance sheath (Medtronic) | The Mitraclip sheath was advanced to the right atrium. A 12-F FlexCath sheath was advanced transeptally through the Mitraclip sheath. The Raptor grasping device was used to grasp the Watchman and retrieve it into the 23-F sheath. | None |
| Mansour et al. ^11^  (2020) | 77M, presented with two ischaemic strokes and left foot pain | Watchman  (24mm) | Abdominal aorta | 1.5 years | Not attempted | n/a | n/a | Owing to significant morbidity in a 77M and a heavily calcified abdominal aorta, device retrieval was not attempted. | The patient died 3 months after discovery |
| Turagam et al.*^12^  (2020) | 73M, asymptomatic, detected on routine post-implant TEE | Amulet (22mm) | Left inferior pulmonary vein ostium | 3 months | Bilateral 6-F CFA access  CFV access for transseptal puncture | Filter Wire EZ cerebral embolic protection devices x 2 (Boston Scientific) | ICE catheter  Prophylactic pigtail catheter insertion into pericardium  12-F Flexcath Advance (Medtronic)  8.5-F Agilis (Abbott)  2.4 x 20mm Raptor grasping device  23-F Micra sheath (Medtronic) | Under ICE and fluoroscopic guidance, a steerable guide sheath was introduced transeptally. A Raptor grasping forceps was advanced to grasp the device. After failure to withdraw the device into a 12-F sheath, it was exchanged for a 23-F Micra sheath and the device was successfully removed. | None |
|  | 72F, asymptomatic, discovered on routine post-implant TEE | Watchman (33mm) | Aortic arch distal to left subclavian ostium, within true lumen of chronic type B aortic dissection | 3 months | Right CFA 20-F access | None | 20-F short sheath  16-F long sheath  27-45mm EN Snare (Merit Medical)  20mm Amplatzer gooseneck snare (Medtronic)  20mm Needle’s Eye Snare (Cook Medical)  7-F Jawz Endomyocardial Biopsy Forceps (Argon Medical Devices) | The device was snared through a 16-F sheath and withdrawn to the abdominal aorta. After failure with a Needle’s Eye snare and endomyocardial biopsy, a Raptor forceps was successful in retrieving the device into the sheath. | None |
|  |  |  |  |  |  |  |  |  |  |
|  |  |  |  |  |  |  |  |  |  |
|  | 79F, discovered on routine post-implant TEE | Watchman  (24mm) | LA | 1 year | 23-F CFA access | 33mm Watchman in Ascending Aorta for Stroke Prevention (WAASP) technique | 12-F Flexcath Advance deflectable sheath (Medtronic)  23-F sheath (Medtronic)  Raptor grasping device | The 12-F deflectable sheath was advanced through a transseptal puncture to the left atrium. The raptor grasping device was advanced through the deflectable sheath to grasp the center of the Watchman both the raptor and the 12-F sheath was withdrawn into the 23-F sheath. | None |
| Al Zahrani et al^13^  (2020) | 74M, asymptomatic, discovered on routine post-implant echocardiogram | Watchman (21mm) | Abdominal aorta | 6 months | Left CFA | None | 14-F sheath (Cook Medical)  Alligator forceps  Prostar XL closure  device (Abbott) | An intracardiac echocardiography (ICE) catheter was advanced through the right internal jugular vein to the inferior vena cava to monitor the aortic lumen during the retrieval procedure. Using a 14-F sheath through the left CFA, the device was captured and removed using alligator forceps. | None at 1 month |
| Tiyerili et al ^14^  (2015) | 79M, patient presented with NYHA class II heart failure symptoms | Amplazter cardiac plug (28mm) | LVOT | 10 weeks | Left CFV | None | 6-F pigtail catheter  14-F sheath (Cook Medical)  20-mm multi-snare catheter | A 6-F pigtail was inserted retrograde in the LVOT to avoid device migration in the ascending aorta. A 14-F sheath was positioned transseptally, and a 20-mm multi-snare catheter device was advanced into the left ventricle for device capture. The 14-F sheath was then advanced through the mitral valve in the left ventricle, which allowed grasping of the distal occluder thread and retrieval into the sheath. | None |
| Peruga et al^15^  (2015) | 67M, asymptomatic, discovered on routine post-implant echocardiogram | Amulet (22mm) | Abdominal aorta | 8 weeks | CFA | None | 16-F steerable sheath (Check-Flo Performer Introducer)  Maslanka grasping forceps  7-F Launcher AR1 (Boston Scientific)  Multi snare | A 16 F steerable sheath was introduced into the femoral artery. A 7-F AR1 guide was introduced via the sheath through which a Maslanka grasping forceps and a Multi snare were advanced. The snare was used to change the orientation of the device enabling the capture of the LAAO device with the grasping forceps. | None |
| Obeid et al^16^  (2014) | 56M, asymptomatic, discovered on routine post-implant TEE | Amplatzer cardiac plug (24mm) | Abdominal aorta | 6 months | Right CFA | None | JR4 diagnostic catheter  Gooseneck snare  14-F femoral sheath | Device captured using a gooseneck snare and pulled into 14-F femoral access sheath | None |
| Perrotta et al^17^  (2013) | 73F, discovered on routine follow-up TTE | AGA cardiac plug (30mm) | LA | 6 weeks | CFV | None | 24-F Mitraclip steerable sheath (Abbott)  30mm snare  2nd snare | The 24-F MitraClip sheath was introduced into the left atrium after transseptal puncture. The waist of the ACP was snared with a 30-mm snare and stabilized by pulling it against the septum. Following this, the distal screw of the ACP device was grasped with a second snare and retracted into  the sheath | None |

*Turagam et al. (2020) reported a case series; individual patient cases are presented in separate rows

NR – not reported, CFA – common femoral artery, CFV – common femoral vein, IAS – interatrial septum, LAA – left atrial appendage, TEE – transesophageal echocardiography, TTE – transthoracic echocardiograph

**References**

1. Siordia JA, Al Hennawi H, Sabri MS, et al. Retrieval of an Embolized Left Atrial Appendage Occluder From the Aortic Arch Using a Transcatheter Retrieval Device. *JACC Case Rep*. Aug 20 2025;30(24):104557. doi:10.1016/j.jaccas.2025.104557

2. Guddeti RR, Sayed A, Seshiah P, Bae R, Garcia S. Percutaneous Removal of Left Atrial Appendage Occlusion Device With a Dedicated Retrieval System. *JACC Case Rep*. Aug 20 2025;30(24):104751. doi:10.1016/j.jaccas.2025.104751

3. Green C, Bonthu S, Oskin TC, Crawford JL. Removal of a third-generation left atrial appendage occlusion device from the pararenal aorta using a novel endovascular technique. *J Vasc Surg Cases Innov Tech*. Dec 2025;11(6):101958. doi:10.1016/j.jvscit.2025.101958

4. Goodyear E, Kunamalla A, Ferro EG, et al. Successful Percutaneous Extraction of a WATCHMAN FLX Device From the Left Ventricular Outflow Tract. *JACC Case Rep*. Jan 23 2025;30(8):103186. doi:10.1016/j.jaccas.2024.103186

5. Nienaber S, Ballmann F, Curio J, Eghbalzadeh K, Sinning JM, Adam M. Percutaneous Retrieval of Embolised Left Atrial Appendage Occluder With the Novel ONO Retrieval Basket. *Can J Cardiol*. Dec 2024;40(12):2414-2416. doi:https://doi.org/10.1016/j.cjca.2024.08.276

6. Wong G, Aman E, Kiaii B, Rogers JH, Singh GD. Transcatheter Extraction of a Migrated Left Atrial Appendage Occluder Device. *JACC Case Rep*. Aug 7 2024;29(15):102443. doi:10.1016/j.jaccas.2024.102443

7. Ferraris F, Millesimo M, Anselmino M, et al. Successful Percutaneous Retrieval of an Embolized Left Atrial Appendage Occluder. *JACC Case Rep*. Dec 21 2022;4(24):101689. doi:10.1016/j.jaccas.2022.101689

8. Cwiek-Rebowska E, Peruga JZ, Szymczyk E, Jankowski L, Kasprzak J. Raiders of the lost... plug. Percutaneous retrieval of embolized left atrial appendage occluder in asymptomatic patient followed by reimplantation of different type device. *Kardiol Pol*. 2021;79(11):1284-1285. doi:10.33963/KP.a2021.0098

9. Agarwal S, Oren JWt, Baig I, et al. Taming a Rogue Watchman. *JACC Cardiovasc Interv*. Jul 26 2021;14(14):e161-e163. doi:10.1016/j.jcin.2021.03.032

10. Maan A, Turagam MK, Dukkipati SR, Reddy VY. Percutaneous Extraction of a Migrated WATCHMAN Device After Seven Months. *J Innov Card Rhythm Manag*. Jul 2021;12(7):4572-4574. doi:10.19102/icrm.2021.120701

11. Mansour MJ, Benic C, Didier R, Noel A, Gilard M, Mansourati J. Late discovery of left atrial appendage occluder device embolization: a case report. *BMC Cardiovasc Disord*. Jun 22 2020;20(1):305. doi:10.1186/s12872-020-01589-9

12. Turagam MK, Neuzil P, Dukkipati SR, et al. Percutaneous Retrieval of Left Atrial Appendage Closure Devices With an Endoscopic Grasping Tool. *JACC Clin Electrophysiol*. Apr 2020;6(4):404-413. doi:10.1016/j.jacep.2019.11.015

13. Al Zahrani YA, Arabi M, Al Harbi AA, Al Dulaigan E, Al Ghamdi A, Al Moaiqel M. Percutaneous Endovascular Retrieval of a Dislodged Left Atrial Appendage Closure Device from the Juxtarenal Aorta. *The Arab Journal of Interventional Radiology*. 2020;4(1):47-48. doi:10.4103/ajir.Ajir_28_19

14. Tiyerili V, Nickenig G, Hammerstingl C. Catch of the day: interventional device retrieval after late embolization of an Amplatzer cardiac plug left atrial appendage occluder. *Clin Res Cardiol*. Dec 2015;104(12):1106-8. doi:10.1007/s00392-015-0887-3

15. Jz P, E Ć-R, E S, L J, J K. Percutaneous Retrieval of Emboli Zed Left Atrial Appendage Occluder Followed by Re-implantation of Different Type Device. *Clinics of Surgery*. 2021;05(05)doi:10.47829/COS.2021.5501

16. Obeid S, Nietlispach F, Luscher TF, Alibegovic J. Percutaneous retrieval of an endothelialized AMPLATZER cardiac plug from the abdominal aorta 6 months after embolization. *Eur Heart J*. Dec 14 2014;35(47):3387. doi:10.1093/eurheartj/ehu361

17. Perrotta L, Bordignon S, Furnkranz A, Chun JK, Eggebrecht H, Schmidt B. Catch me if you can: transseptal retrieval of a dislodged left atrial appendage occluder. *Circ Arrhythm Electrophysiol*. Aug 2013;6(4):e64. doi:10.1161/CIRCEP.113.000671
